# Full-endoscopic decompression surgery in the treatment of elderly patients with degenerative lumbar spinal stenosis

**DOI:** 10.3389/fsurg.2025.1582877

**Published:** 2025-06-16

**Authors:** Shangjv Gao, Lifang Shi, Can Cao, Jingchao Wei, Wenjie Lv, Wenyi Li

**Affiliations:** Department of Orthopedics, Hebei General Hospital, Shijiazhuang, Hebei, China

**Keywords:** lumbar spinal stenosis, minimally invasive, full-endoscopic, low back pain, elderly

## Abstract

**Objective:**

To introduce the technical protocol of the full-endoscopic decompression surgery (FEDS) in the treatment of elderly patients with degenerative lumbar spinal stenosis (DLSS) and evaluate its clinical efficacy compared with posterior transforaminal lumbar interbody fusion (PTLIF).

**Methods:**

A retrospective study was conducted on 82 elderly patients (aged ≥70 years) with DLSS, including 45 patients who underwent FEDS (FEDS group) and 37 patients who underwent PTLIF (PTLIF group). General data including age, sex, American Society of Anesthesiologists (ASA) classification, surgical segment, preoperative visual analogue scale (VAS) for low back pain and leg pain, and preoperative Oswestry Disability Index (ODI) were compared between the two groups. VAS for low back pain, VAS for leg pain, and ODI were recorded at 6 weeks, 6 months, and the last follow-up after surgery. Operation-related parameters such as operation time and length of hospital stay were recorded. Early and late complications were also compared between the two groups to assess their safety and efficacy.

**Results:**

The average age of patients in the FEDS group was 75.6 years, older than that in the PTLIF group (74.1 years, *P* = 0.037). The preoperative VAS for low back pain was lower in the FEDS group compared to the PTLIF group (*P* = 0.022). There were no significant differences between the two groups in terms of sex, ASA classification, surgical segment, preoperative VAS for leg pain, and preoperative ODI. The follow-up period was 17.0 ± 3.7 months (range 12–30 months). Significant improvements in VAS for low back pain, VAS for leg pain, and ODI were observed during follow-ups compared to preoperative values. The FEDS group had shorter operation time and length of hospital stay compared to the PTLIF group (both *P* < 0.001). There were no significant differences in early and late complications between the two groups, although the types of complications differed.

**Conclusion:**

FEDS is as effective as PTLIF in the treatment of elderly patients with DLSS.

## 1 Introduction

Degenerative lumbar spinal stenosis (DLSS) is a prevalent and disabling cause of low back and leg pain in older persons. Its incidence is highly correlated with age, with a prevalence of approximately 11% in US adults (1). It often has a long course and significantly impacts on patients' daily lives. Surgical treatment should be considered in patients experiencing severe pain affecting daily activities, evident signs of neurological damage, severe intermittent claudication (walking distance <500 meters), and ineffective conservative treatment lasting more than three months (2).

Surgical treatment for elderly patients presents a significant challenge for spine surgeons because these patients often have comorbidities such as osteoporosis, muscle degeneration, and comorbidities including cardiovascular disease, diabetes, and respiratory system disorders. When the surgical technique is selected for these patients, in addition to surgical efficacy, the risks of surgical trauma and anesthesia are also crucial considerations (3–5).

Posterior transforaminal lumbar interbody fusion (PTLIF) can achieve sufficient decompression of the spinal canal and good efficacy for various types of lumbar spinal stenosis, making it one of the most widely used and effective surgical technique (6, 7). However, it is associated with long operation time, significant trauma, and certain perioperative complications such as internal fixation failure, non-fusion, and adjacent segment degeneration (ASD) (8–10). Additionally, many patients are unable to tolerate general anesthesia due to contraindications such as poor pulmonary function and unstable angina pectoris, thereby missing the opportunity for surgical decompression (11).

For elderly patients with DLSS, the full-endoscopic decompression surgery (FEDS) under local anesthesia has become a promising surgical option (12). This study aims to introduce the FEDS technical protocol and evaluate its clinical efficacy compared to traditional PTLIF surgery, providing a minimally invasive, safe, and effective treatment option for such patients.

## 2 Materials and methods

### 2.1 Patient characteristics

This study was a retrospective study approved by the hospital ethics committee. Elderly patients (age ≥70 years) diagnosed with DLSS and undergoing surgical treatment in the Department of Orthopedics at Hebei General Hospital from January 2018 to December 2020 were included. The inclusion criteria were as follows: (1) Age ≥70 years; (2) Lower limbs radicular pain or intermittent claudication with walking distance <500 meters; (3) Radiographic findings consistent with clinical symptoms on CT or MRI; (4) Ineffectiveness of conservative treatment or signs of neurological impairment such as motor and sensory deficits; (5) Single-level surgery was performed; (6) Follow-up period of at least 12 months. The exclusion criteria were as follows: (1) Lumbar spondylolisthesis >1 degree or segmental instability of the lumbar spine according to the diagnostic criteria of White and Panjabi (13); (2) Concurrent spinal deformity, infection, or tumor; (3) Psychiatric disorders.

After screening, 82 patients were included, including 50 males and 32 females, with a mean age of 74.9 ± 3.3 years (range 70–82 years). Patients were divided into two groups: the FEDS group (45 cases) underwent full-endoscopic decompression surgery, and the PTLIF group (37 cases) underwent posterior transforaminal lumbar interbody fusion surgery. The surgical segment was determined based on clinical and radiographic findings preoperatively. If the segment was unclear, selective nerve root block (SNRB) was performed to assist in identifying the responsible segment (14–16). Patients underwent relevant imaging examinations and laboratory tests based on comorbidities. If necessary, consultations with doctors in relevant specialties were needed.

### 2.2 Surgical methods

#### 2.2.1 FEDS technical protocol

Anesthesia was administered as monitored anesthesia care (MAC) with local anesthesia. An anesthesiologist monitored vital signs including heart rhythm, blood pressure, respiration, and pulse oximetry during surgery. Wakeful analgesia was achieved using a 4 μg/ml concentration of dexmedetomidine, administered intravenously at a dose of 1 μg/kg via a micro-infusion pump. The surgical approach (transforaminal or interlaminar) was chosen based on the location of stenosis, surgical segment, and clinical symptoms. Generally, the transforaminal approach was selected for lateral recess or foraminal stenosis, while the interlaminar approach was chosen for central canal stenosis.

##### 2.2.1.1 Transforaminal approach

**Figure 1:**
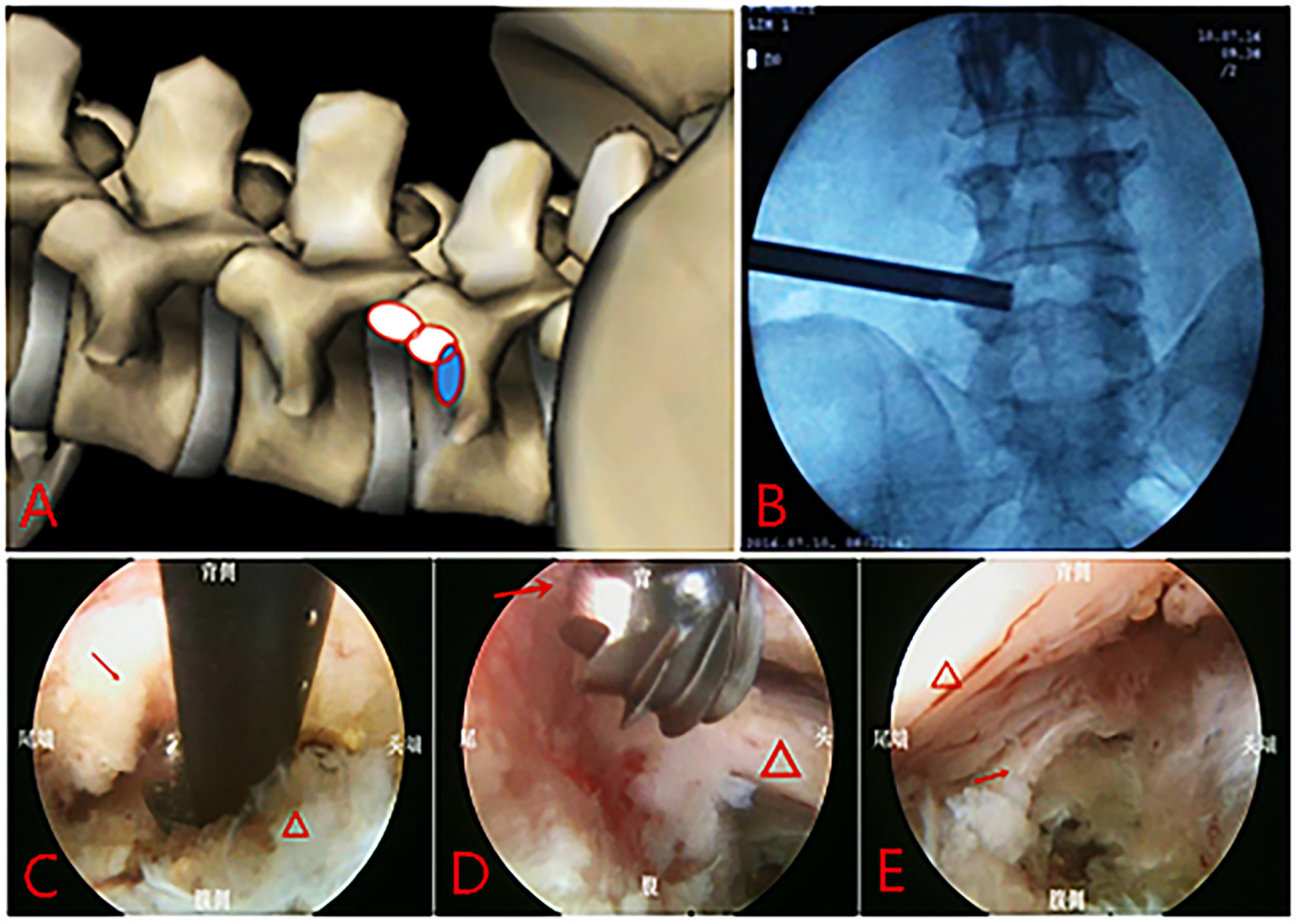
Full-endoscopic decompression surgery via transforaminal approach: **(A)** range of the foraminoplasty; **(B)** position of the trephine on the AP fluoroscopy image. **(C)** Resection of upper articular process with Kerrison rongeur under endoscopy, “→” shows the ventralside of superior facet joint. “△” shows the disc; **(D)** Remove the osteophytes of posterior vertebral edge using high-speed burr, “→” shows the steophytes. “△” shows the nerve root. **(E)** The nerve root and dural sac after decompression, “→” shows the posterior longitudinal ligaments. “△” shows the nerve root and dural sac after decompression.

The patient was placed in a prone position with the abdomen suspended, knees and hips slightly flexed. Under C-arm fluoroscopy in anteroposterior and lateral views, the puncture path was determined using a wire and marked on the skin. Under fluoroscopic guidance, the needle was punctured to the Kambin triangle via the transforaminal approach. The sheath was inserted under wire guidance, and the intervertebral foramen was enlarged using a trephine under fluoroscopic guidance. The expanded area included the tip, body, and root of the superior articular process, even part of the pedicle. After foraminoplasty (Figures 1A,B), the working channel was inserted, and decompression were performed under endoscopy using a Kerrison rongeur and a high-speed burr (Figure 1C). The ligamentum flavum on the medial aspect of the superior articular process was excised and discectomy were performed (Figure 1D). If foraminal stenosis was observed in preoperative imaging examination, the ligamentum flavum and osteophytes at the distal end of the exiting nerve root were excised to decompress the nerve. In addition, contralateral decompression was performed via the intervertebral space if the patient had contralateral symptoms. After the ipsilateral discectomy, the endoscope was lowed to reduce its horizontal angle, then angled forceps was used to perform the contralateral discectomy below the posterior longitudinal ligament. The entire discectomy increased the ventral space of the spinal canal. The surgical endpoint was achieved when the compressed nerve root and dural sac returned to their normal positions and pulsations were observed with changes in water pressure (Figure 1E).

##### 2.2.1.2 Interlaminar approach

**Figure 2:**
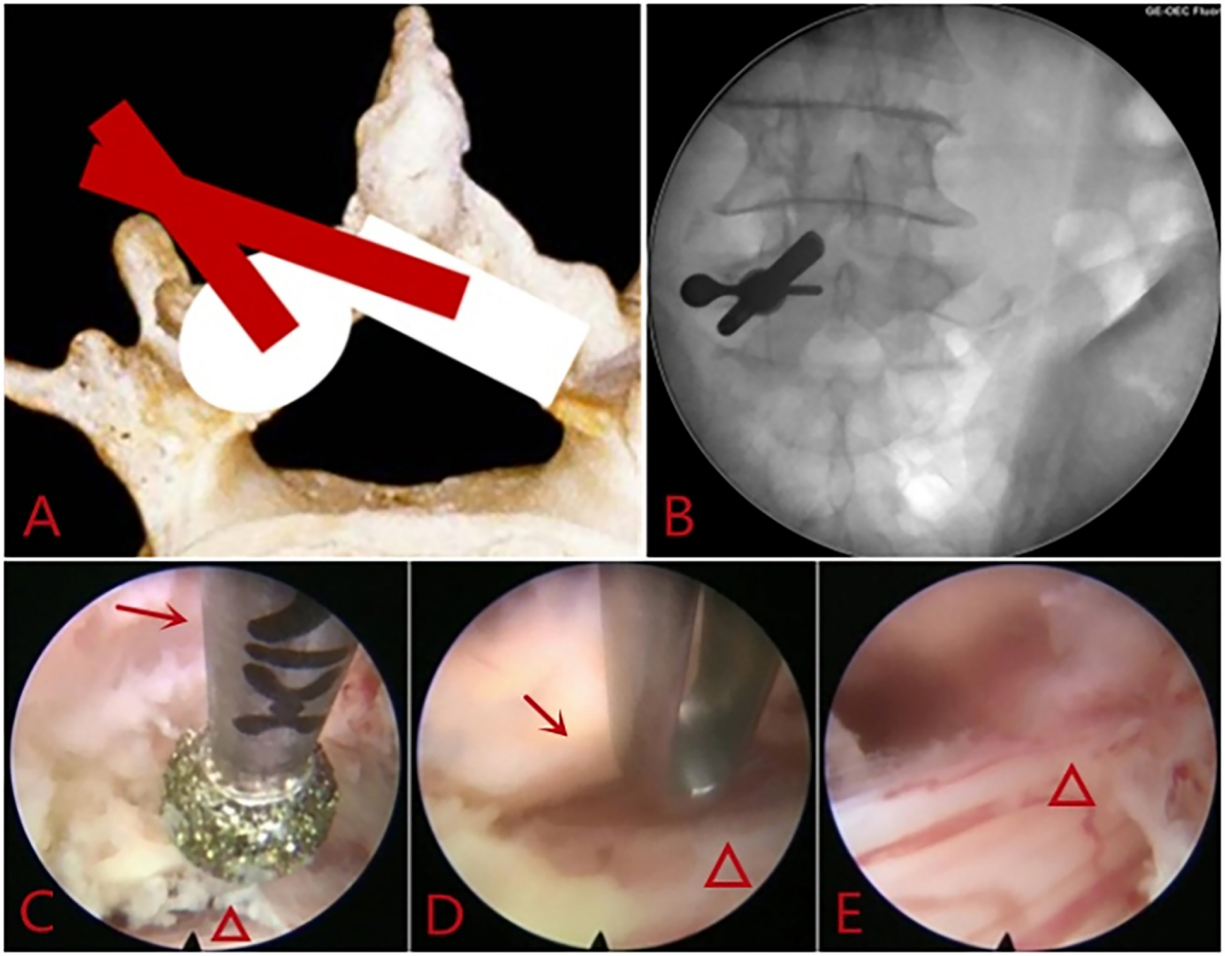
Full-endoscopic decompression surgery via interlaminar approach: **(A)** range of the decompression. **(B)** Location of the working sheath on the AP fluoroscopy image. **(C)** Contralateral Laminectomy using the high- speed burr, “→” shows the ventralside of the lamina. “△” shows the dural sac. **(D)** Resection of the contralateral hypertrophic ligamentum flavum, “→” shows the hypertrophic ligamentum flavum. “△” shows the dural sac. **(E)** Nerve roots and dural sac after decompression.

The decompression range was planned before surgery based on the patient's symptoms (Figure 2A). Unilateral laminectomy with bilateral decompression (ULBD) or unilateral decompression was determined. The starting point for decompression was selected at the junction between the root of spinous process and the lamina (Figure 2B). After anesthesia and punch, the working channel was inserted, and after clearing the soft tissue from the lamina and ligamentum flavum surfaces, the surgical field on the operative side was fully exposed. Starting from the lower edge of the lamina, laminectomy was performed using a Kerrison rongeur and a high-speed burr. The longitudinal decompression range extended to the proximal and the distal end of the ligamentum flavum. The outward decompression range extended to the outer edge of the nerve root. If bilateral symptoms was present, “over-the-top” technique (17) was utilized to remove part of the bone from the base of the spinous process (Figures 2C,D). After decompression, a significant increase in the volume of the spinal canal was observed, with ample space around the compressed nerve roots and dural sac (Figure 2E).

No drainage device was required after the operation. The patient should rest in bed on the day of surgery to prevent bleeding. On the first day after surgery, the patient could start to move with the protection of a waistband. The waistband should be worn for 6 weeks. Physical labor and intense sports activities should be avoided for 3 months.

#### 2.2.2 PTLIF technical protocol

After general anesthesia, the patient was placed in the prone position. A posterior midline incision was made, and the paravertebral muscles were dissected along the spinous process to expose the lamina and facet joints. After confirming the target segment under fluoroscopy, pedicle screws were placed. The laminectomy of the responsible segment and the excision of the thickened ligamentum flavum are performed. After hemostasis, the Kambin triangle was exposed. Discectomy and interbody fusion were performed under careful protection to the nerve. After meticulous hemostasis, the wound was closed, leaving one drainage tube in the surgical site.

### 2.3 Clinical efficacy indicators

Preoperative physical condition and comorbidities were assessed using the American Society of Anesthesiologists (ASA) classification (18). Operation time and length of hospital stay were used to evaluate perioperative conditions. Due to the inability to calculate blood loss in the FEDS group, a comparison of blood loss between the two groups was not conducted. The visual analogue scale (VAS) for low back pain and leg pain was used to assess postoperative pain levels at each timepoints during follow-up. The Oswestry Disability Index (ODI) (19) was utilized to evaluate postoperative function at 6 weeks, 6 months, and last follow-up after surgery. Patient satisfaction was assessed using the Odom criteria (20) at the last follow-up, categorized as excellent (all preoperative symptoms greatly relieved), good (minor residual symptoms), fair (partial relief of preoperative symptoms), or poor (no improvement or worsening of preoperative symptoms). Additionally, complications of both surgical procedures were compared, including early complications such as nerve injury, cerebrospinal fluid leakage, epidural hematoma, surgical site infection, heart failure, pulmonary infection, and urinary tract infection, as well as late complications such as internal fixation failure, recurrent disc herniation, adjacent segment degeneration (ASD), and non-fusion.

### 2.4 Statistical analysis

SPSS 23.0 (Statistical Product and Service Solutions software version 23.0; SPSS, Chicago, IL) was used for data analysis. Depending on the nature of the data, the age, VAS and ODI were represented by mean ± standard deviation, and sex and ASA classification were represented by frequency (rate). For continuous variables with homogeneity of variance, independent sample t-tests were used for comparison, while for variables without homogeneity of variance, the Mann–Whitney *U* test was used. Paired sample *t*-test was used for comparison of continuous data before and after surgery. For categorical data, either the chi-square test or Fisher's exact test was used for comparison, with a significance level set at *P* < 0.05 indicating statistical difference.

## 3 Result

Baseline data of the two groups of patients: there were 45 cases in the FEDS group and 37 cases in the PTLIF group. The common comorbidities in both groups were hypertension, arrhythmia, coronary heart disease, chronic obstructive pulmonary disease, diabetes, and chronic kidney disease. There were no significant differences between the two groups in terms of sex, ASA classification, surgical segment, and follow-up period (all *P* > 0.05). However, the age of the FEDS group was higher than that of the PTLIF group, with statistical significance (*P* = 0.037) (Table 1).

**Table 1:** Preoperative clinical characteristics of two groups.

Variables	FEDS group (*n* = 45)	PTLIF group (*n* = 37)	X^2^/t	*P*
Age (years)	75.6 ± 3.4	74.1 ± 2.9	2.12	0.037
Sex			0.07	0.799
Male (%)	28 (62.2)	22 (29.5)		
Female (%)	17 (37.8)	15 (40.5)		
Surgical segment			2.05	0.563
L2-3	3	3		
L3-4	7	10		
L4-5	24	15		
L5-S1	11	9		
ASA classification			2.13	0.345
Ⅱ	11	13		
Ⅲ	30	23		
Ⅳ	4	1		
Preoperative				
VAS for low back pain	3.3 ± 1.3	4.0 ± 1.4	−2.34	0.022
VAS for leg pain	5.9 ± 1.7	5.2 ± 1.5	1.97	0.052
ODI	64.1 ± 9.6	60.5 ± 6.3	1.96	0.054
Follow-up period (months)	17.6 ± 3.8	16.2 ± 3.5	1.79	0.077

ASA, American Society of Anesthesiologists; VAS, visual analog scale; ODI, Oswestry disability index.

There were no significant differences between the two groups in preoperative VAS for leg pain and ODI (both *P* > 0.05). However, preoperative VAS for low back pain in the PTLIF group was higher than that in the FEDS group, with statistical significance (*P* = 0.022).

Both groups of patients underwent surgery successfully. The operation time for the FEDS group was 73.2 ± 20.5 min (range 40–120 min), with the length of hospital stay of 9.4 ± 2.7 days (range 4–16 days). In contrast, the operation time for the PTLIF group was 149.7 ± 26.4 min (range 100–200 min), with the length of hospital stay of 13.0 ± 3.4 days (range 6–19 days). The operation time and length of hospital stay were significantly lower in the FEDS group compared to the PTLIF group, with a statistically significant difference (both *P* < 0.001).

**Figure 3:**
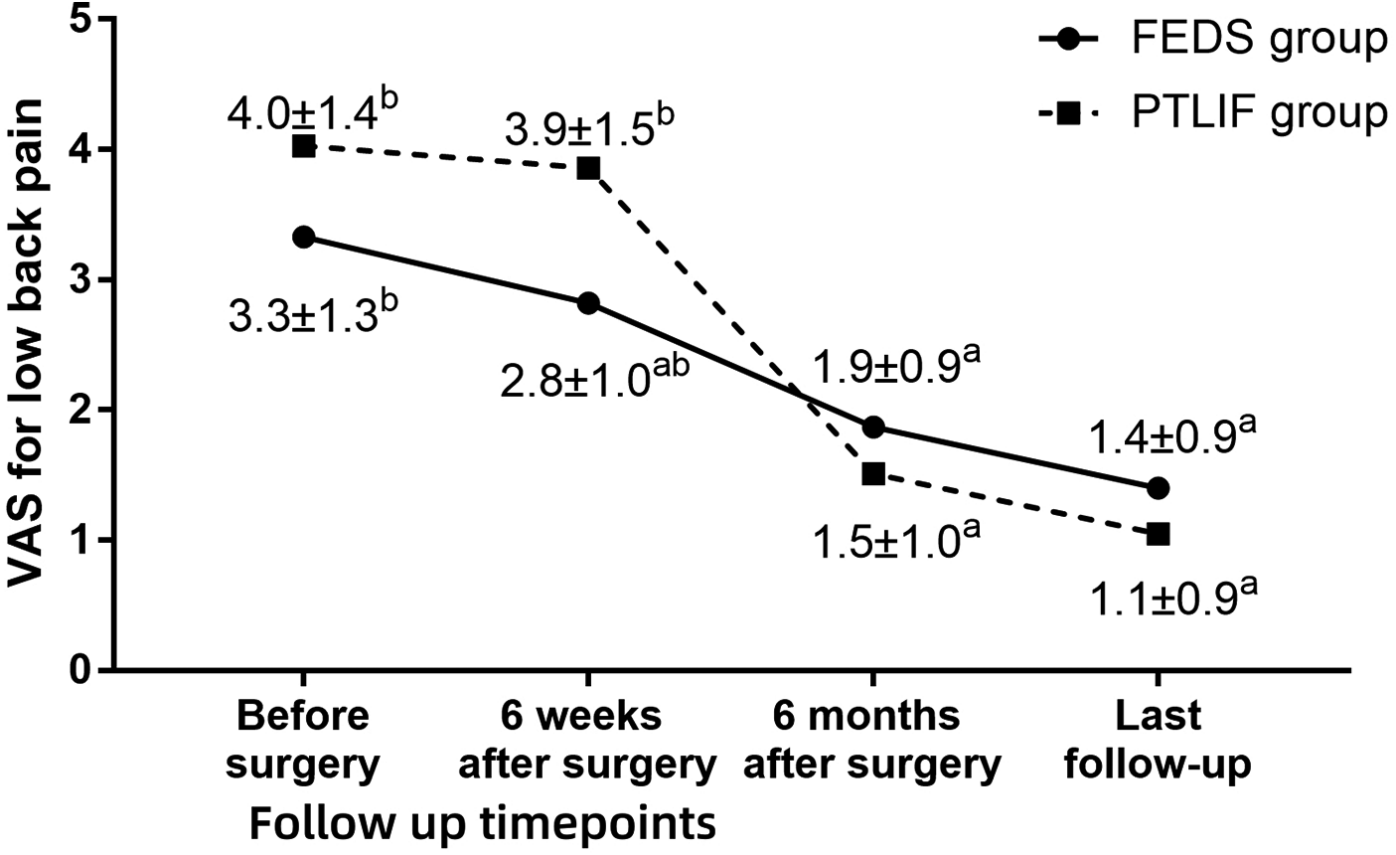
Visual analogue scale (VAS) for low back pain in two groups at each timepoint during follow-up. ^a^ *P* < 0.05 compared with preoperative values. ^b^ *P* < 0.05 between two group at same timepoint.

**Figure 4:**
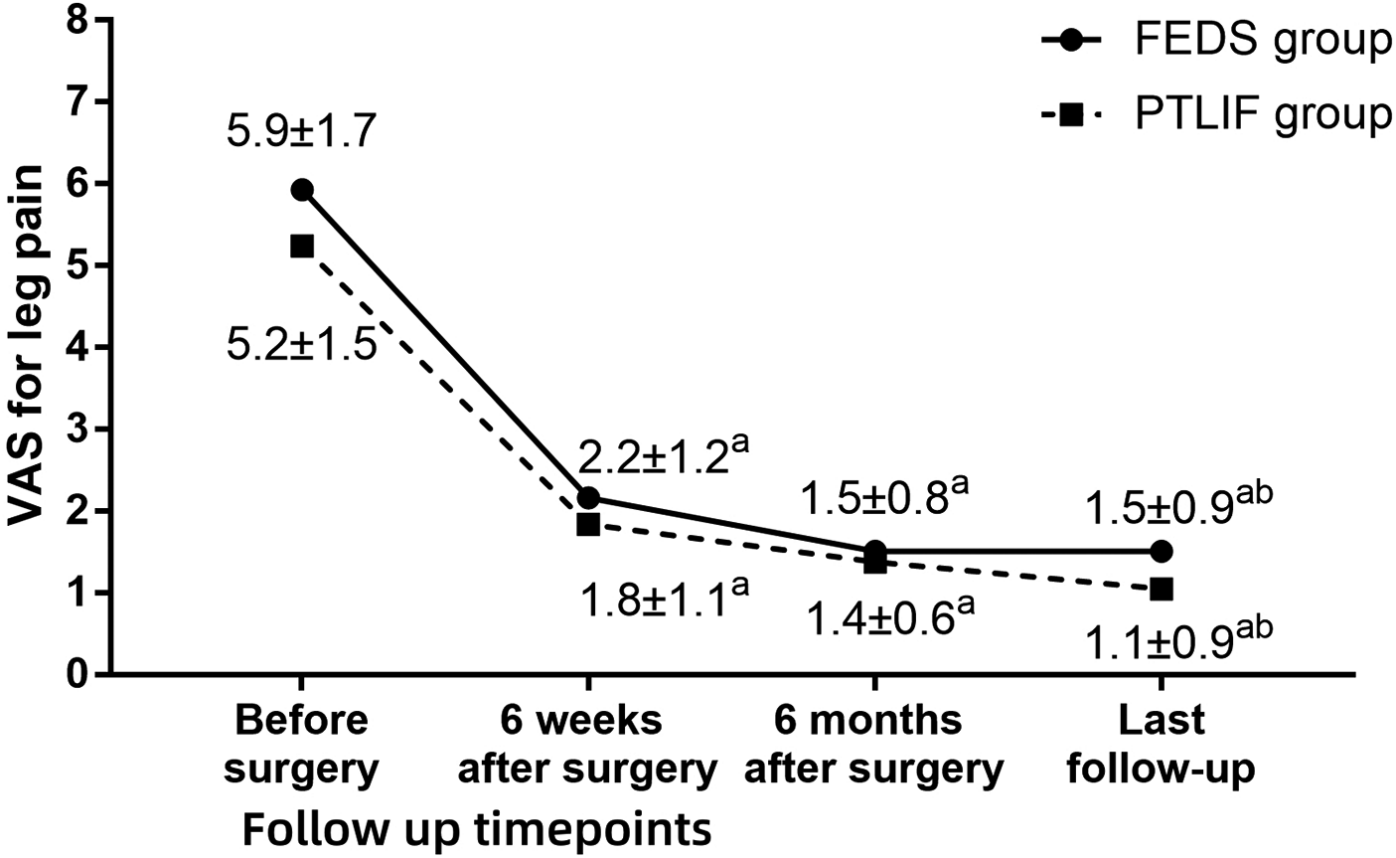
Visual analogue scale (VAS) for leg pain in two groups at each timepoint during follow-up. ^a^ *P* < 0.05 compared with preoperative values. ^b^ *P* < 0.05 between two group at same timepoint.

**Figure 5:**
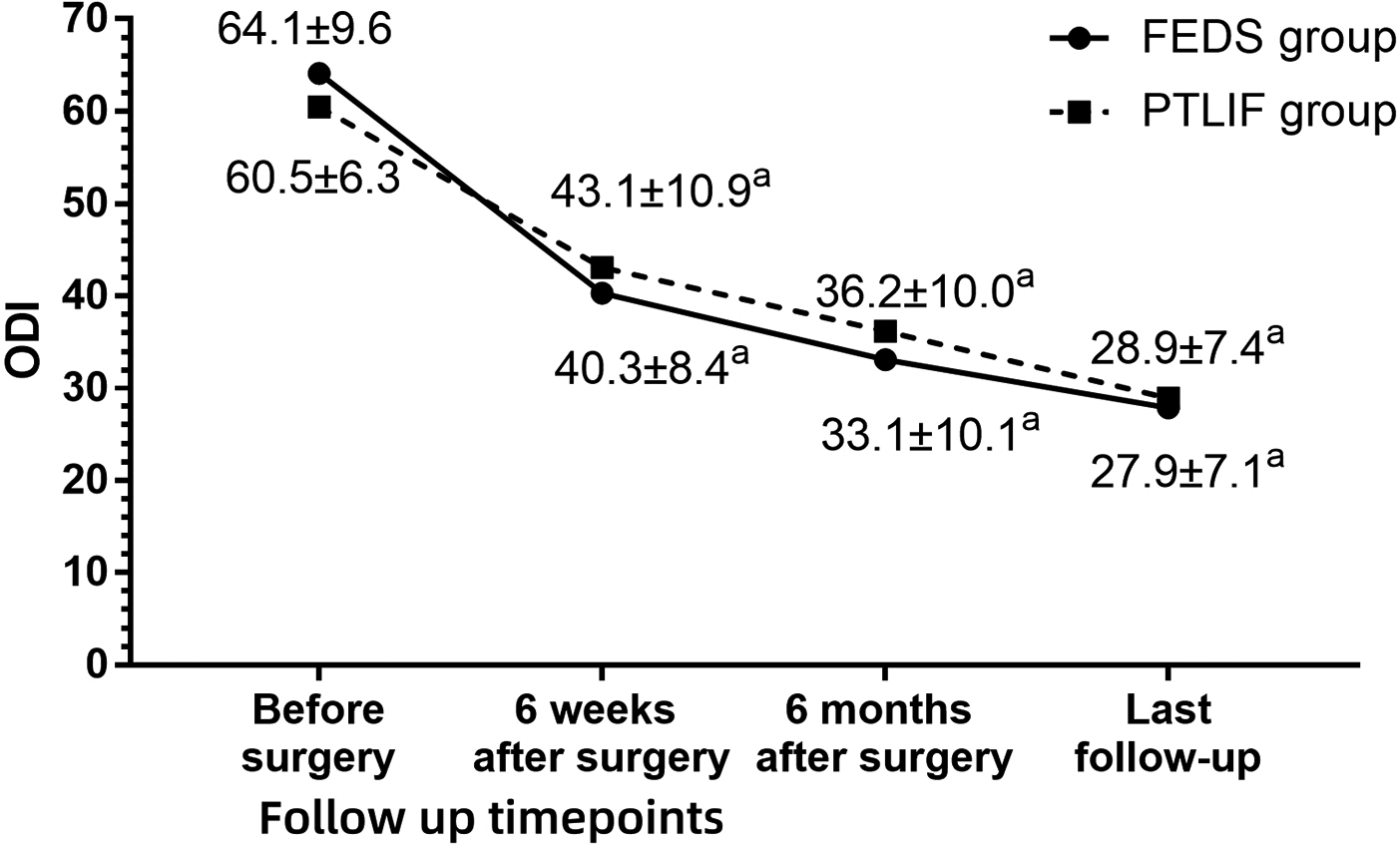
Oswestry disability Index (ODI) in two groups at each timepoint during follow-up. ^a^ *P* < 0.05 compared with preoperative values. ^b^ *P* < 0.05 between two group at same timepoint.

The follow-up period was 17.0 ± 3.7 months (range 12–30 months). At 6 weeks after surgery, the VAS of low back pain in the PTLIF group was lower than that before surgery, but the difference was not statistically significant (*P* = 0.679). Except for it, both groups at different points during follow-up showed significant improvement in VAS for low back pain, VAS for leg pain, and ODI compared to preoperative values (all *P* < 0.05). Compared with PTLIF group, the VAS for low back pain at 6 weeks postoperatively was lower in the FEDS group than in the PTLIF group (*P* < 0.001), and the VAS for leg pain at the last follow-up was higher in the FEDS group than in the PTLIF group (*P* = 0.027). Except for them, there were no statistically significant differences in the other indicators at various time points during follow-up between the two groups (all *P* > 0.05) (Figures 3–5). There was no significant difference in patient satisfaction with the treatment between the two groups (*P* = 0.355) (Table 2).

**Table 2:** Odom criteria of the two groups at the last follow-up.

Variables	FEDS group (*n* = 45)	PTLIF group (*n* = 37)	X^2^	*P*
Odom criteria			3.25	0.355
Excellent	10	14		
Good	27	20		
Fair	5	2		
Poor	3	1		

Both groups had no perioperative mortality cases. In the FEDS group: One patient developed acute left heart failure on the night of surgery, but improved after treatment in the cardiovascular department; one case experienced intraoperative nerve injury, resulting in a decrease in extensor digitorum strength from grade 4 preoperatively to grade 1 postoperatively. Another patient had intraoperative cerebrospinal fluid leakage, which was not specifically treated, and showed no signs of wound healing problem. One patient experienced recurrent disc herniation two months after surgery and was relieved after undergoing another FEDS procedure. In the PTLIF group: One case experienced cerebrospinal fluid leakage, which was repaired intraoperatively, and the patient had delayed removal of the drainage tube but achieved good wound healing postoperatively. Postoperative pulmonary infection, urinary tract infection, and acute heart failure occurred in one case respectively, all of which improved after internal medicine treatment. At the last follow-up, one case had cage subsidence and one case had non-fusion, but both patients did not experience significant low back pain or leg pain symptoms and did not undergo further treatment. Two cases in the PTLIF group developed ASD, one case underwent surgery again, and the other case improved after conservative treatment. The incidence of complications did not differ significantly between the two groups (*P* > 0.05), but there were significant differences in the types of complications (Table 3).

**Table 3:** Complications of two surgical methods.

Variables	FEDS group (*n* = 45)	PTLIF group (*n* = 37)	X^2^	*P*
Complications (%)	4 (8.9)	8 (21.6)	2.64	0.105
Early complications	3 (6.7)	4 (10.8)	0.45	0.504
Nerve root injury	1	–		
Cerebrospinal fluid leakage	1	1		
Acute left heart failure	1	1		
Pulmonary infection	–	1		
Urinary tract infection	–	1		
Late complications	1 (2.2)	4 (10.8)	2.62	0.106
Recurrent disc herniation	1	–		
Cage subsidence	–	1		
Adjacent segment degeneration	–	2		
Non-union	–	1		

## 4 Discussion

In this study, there was a difference in age between the two groups of patients, with the FEDS group having a slightly higher mean age (75.6 compared to 74.1 years). The reason may be that elderly patients often have multiple comorbidities and may also have more severe osteoporosis, making them less tolerant to PTLIF surgery and anesthesia. Moreover, the probability of internal fixation failure is higher in older patients, leading surgeons to exercise greater caution when selecting PTLIF surgery. Although there was no statistically significant difference in preoperative ASA classification between the two groups, the FEDS group had 4 cases of ASA class IV compared to 1 case in the PTLIF group. This suggests that the overall condition of patients in the FEDS group may have been more severe than in the PTLIF group, possibly indicating that some patients who could not tolerate general anesthesia for PTLIF surgery had no choice but to undergo FEDS.

Patients undergoing PTLIF surgery had higher preoperative VAS for low back pain than those in the FEDS group, indicating that these patients may have had underlying lumbar instability or stable spondylolisthesis even though they did not meet the diagnostic criteria set by White and Panjabi (13). At 6 weeks after surgery, VAS for low back pain in the FEDS group were lower than those in the PTLIF group, possibly due to the slightly lower preoperative low back pain in the FEDS group. Additionally, PTLIF surgery involves greater trauma and more extensive soft tissue dissection and bone removal. However, during subsequent follow-up, there was no significant statistical difference in VAS for low back pain between the two groups.

In the FEDS group, two minimally invasive surgical approaches, transforaminal and interlaminar, were employed. Liang et al. compared the efficacy of transforaminal and interlaminar approaches for the treatment of lumbar spinal stenosis, and found that both approaches had comparable VAS for low back pain, but VAS for leg pain in the transforaminal approach was higher than that in the interlaminar approach postoperatively (21). In this study, we observed similar results of VAS of low back pain and leg pain, which indicating that both surgical methods can adequately decompress the nerve roots. At the last follow-up, both groups showed significant improvement in ODI, meeting the basic requirements for daily life. Based on the postoperative improvement in VAS and ODI, both surgical methods achieved high patient satisfaction.

The FEDS group exhibited significant advantages in terms of operation time and length of hospital stay, attributed to the smaller surgical trauma associated with minimally invasive procedures. Although there were no statistically significant differences, FEDS patients experienced fewer complications compared to the PTLIF group, possibly due to shorter operation time, anesthesia methods, and reduced fluid infusion. Spinal endoscopic surgery involves local anesthesia with monitoring, while open spinal decompression fusion surgery typically requires general anesthesia. Local anesthesia has minimal systemic effects on the respiratory and circulatory systems, with lower anesthesia-related risks compared to spinal and general anesthesia (22). Local regional anesthesia offers several advantages, including higher safety, avoidance of postoperative nausea and vomiting associated with general anesthesia, shorter recovery time, absence of urinary catheter retention during surgery, and reduced risk of urinary-related complications (23). In this study, the local anesthesia used in the FEDS group was not strictly localized; after puncture completion, ropivacaine was injected into the extradural space via the puncture needle. Ropivacaine is a long-acting local anesthetic with a motor-sensory separation effect commonly used in painless childbirth. It allows monitoring of the patient's muscle strength and response to severe nerve stimulation during surgery. General anesthesia under intubation has a greater impact on the patient's respiratory and circulatory systems. In elderly patients with comorbidities, their inadequate cardiopulmonary reserve makes them susceptible to perioperative complications due to the impact on airway and circulation, changes in vascular tension, and poor compensatory ability under anesthesia. In this study, one case of pneumonia and urinary tract infection occurred in the PTLIF group, possibly related to endotracheal intubation and urinary catheterization. Although one case of acute heart failure occurred in each group, the overall preoperative condition of the FEDS group was poorer than that of the PTLIF group. Machado et al. reported a perioperative complication rate of nearly 4% in elderly patients with lumbar spinal stenosis undergoing surgical treatment, with a higher incidence of complications in patients undergoing traditional bone fusion internal fixation surgery compared to those undergoing simple decompression surgery (24).

In this study, SNRB was used to assist in disease diagnosis and determination of the responsible segment. In cases where clinical manifestations did not clearly localize the nerve or when imaging data showed varying degrees of spinal stenosis across multiple segments, SNRB was employed to delineate the responsible segment. SNRB offers significant advantages in determining the responsible segment of lumbar spine diseases. However, it also carries certain false positive and false negative rates, with studies indicating an accuracy rate of 73%. False positives and negatives may occur due to inaccurate injection sites or epidural diffusion (25). Clinical judgment should combine both the patient's clinical and imaging presentations. In our study, the local anesthetic used was 1% lidocaine 1 ml, administered by experienced spinal surgeons under C-arm guidance. Positive results were defined as a ≥60% reduction in preoperative lower limb symptoms lasting over 2 h (26). Following the procedure, patients immediately walked under the protection of medical staff and simulated positions or movements that typically induced symptoms. If symptom relief was less than 60%, another segmental SNRB was conducted after an interval of more than 12 h.

In elderly patients, the intervertebral disc has lower water and collagen content, increasing the risk of recurrence postoperatively. Therefore, loose nucleus pulposus within the disc should be removed as much as possible, especially at the ventral aspect of the disc where the nerve root and annulus fibrosus rupture, to reduce the recurrence rate. During interlaminar decompression surgery, the outer range of decompression extends to the outer edge of the nerve root, with the upper and lower boundaries being the superior and inferior limits of the ligamentum flavum. During ULBD, exposure and decompression of the contralateral recess should be ensured.

Limitations of this study include its retrospective nature, inability to randomize cases, limited sample size, and baseline differences in age and preoperative low back pain between the two groups. Additionally, this study did not incorporate factors such as osteoporosis and body mass index into the analysis of influencing factors. Since not all patients underwent bone mineral density, the degree of osteoporosis before surgery was not compared between the two groups.

## 5 Conclusion

FEDS demonstrates efficacy comparable to PTLIF in the treatment of elderly patients with degenerative lumbar spinal stenosis. This technique provides an alternative treatment option for elderly patients with poor physical conditions.

## Data Availability

The raw data supporting the conclusions of this article will be made available by the authors, without undue reservation.
